# Spinal Accessory Nerve Mononeuropathy Following Trapezius Lipoma Resection: A Case Report

**DOI:** 10.7759/cureus.80134

**Published:** 2025-03-06

**Authors:** Michael A Serra-Jovenich, Adam T Friedman, Zachary Noll, Trina Lisko

**Affiliations:** 1 Department of Physical Medicine and Rehabilitation, Inspira Medical Center Mullica Hill, Mullica Hill, USA; 2 Department of Rehabilitation Medicine, NeuroMusculoskeletal Institute, Rowan-Virtua School of Osteopathic Medicine, Sewell, USA

**Keywords:** lipoma, spinal accessory nerve, spinal accessory nerve injury, spinal accessory nerve palsy, trapezius lipoma, trapezius lipoma resection

## Abstract

The spinal accessory nerve (SAN) is crucial for the motor function of the trapezius and sternocleidomastoid muscles, playing a significant role in scapular stability and upper limb mobility. While SAN injuries are commonly associated with iatrogenic causes such as lymph node biopsies, neck dissections, and posterior cervical trauma, injury following trapezius lipoma resection has not been well-documented. Given the SAN's superficial location and proximity to surgical fields in the posterior cervical triangle, it is vulnerable to inadvertent injury during tumor excision. To the best of our knowledge, this case report presents the first documented instance of isolated SAN mononeuropathy following trapezius lipoma resection and underscores the importance of early recognition, diagnosis, and management.

A 54-year-old male developed SAN mononeuropathy following an elective surgical removal of a deep right-sided trapezius lipoma. One week following resection, the patient exhibited new-onset paresthesias in the right upper extremity, scapular dyskinesis, shoulder weakness, and lateral scapular winging. Prior electrodiagnostic (EDX) studies for different complaints showed no signs of peripheral nerve injury. Given the new presentation, repeat EDX were performed six months postoperatively, revealing severe focal SAN neuropathy with ongoing active denervation. The findings suggest direct or traction-related SAN injury rather than an isolated muscular pathology. Given the course of symptoms, a referral to a peripheral nerve surgeon was warranted for recommendations regarding further management.

SAN mononeuropathy following lipoma resection is an important and uncommon complication. Most SAN injuries result from iatrogenic causes, such as neck dissections and lymph node biopsies or lateral neck trauma. The SAN’s close proximity to the surgical site makes it susceptible to iatrogenic injury in posterior cervical triangle procedures. This case highlights the importance of identifying lipoma location and recognizing subtle nerve injuries often overlooked without thorough postoperative assessment. Clinical signs include lateral scapular winging during active external rotation, vague shoulder pain, limitations in range of motion, and potential muscle atrophy. In this case, resection of a deeply situated trapezius lipoma led to SAN dysfunction within one week of surgery. Clinical suspicion, combined with examination and confirmation through EDX, highlights the SAN's vulnerability in this region. SAN injury outcomes vary widely, with treatment options ranging from conservative management to surgical interventions such as nerve grafting, nerve repair, or Eden-Lange muscle transfer. Successful recovery is more likely when repairs are performed early, ideally within seven months, with poorer results after 20 months.

This case emphasizes the potential for SAN injury during deep trapezius lipoma resection. Early recognition through physical exam and EDX is essential for distinguishing SAN mononeuropathy from other shoulder dysfunctions, as prompt diagnosis improves functional outcomes. Additionally, preoperative nerve mapping is crucial when operating near critical neural structures, stressing the importance of intraoperative nerve monitoring to reduce the risk of injury. Further research is needed to better understand SAN injury incidence in posterior cervical and scapular surgeries and to develop standardized management guidelines.

## Introduction

The spinal accessory nerve (SAN) plays a critical role in the motor function of the trapezius and sternocleidomastoid muscles, contributing to scapular stabilization and movements such as shoulder elevation and head rotation [1,2]. Lesions to the SAN can result in significant functional impairment, including weakness in shoulder shrugging, difficulty with overhead activities, scapular winging, and potentially result in chronic pain [3]. Injury to the SAN is most often associated with iatrogenic injuries during surgical procedures, such as lymph node biopsies and neck dissections, or trauma to the posterior cervical region [4]. However, its occurrence following trapezius lipoma resection is yet to be defined.

Lipomas are the most common benign mesenchymal tumors, often presenting as painless, slow-growing masses. Although generally harmless, their location near neurovascular structures can pose surgical challenges. The SAN’s superficial course in the posterior triangle of the neck and its deep penetration into the trapezius muscle make it particularly vulnerable to injury during tumor excision [5].

To date, only one documented case of SAN injury following lipoma removal exists; however, the exact location of the lipoma was not specified in that report [4]. To the best of our knowledge, this case report presents the first published case of isolated SAN injury following a trapezius lipoma resection. By detailing the clinical presentation, diagnostic approach, and management strategy, this study aims to raise awareness among physicians about potential SAN injury. Understanding the delicate anatomy of the SAN and implementing nerve-sparing surgical approaches are essential to preventing iatrogenic injury, highlighting the importance of early recognition and intervention to optimize patient outcomes [6,7].

## Case presentation

**Figure 1:**
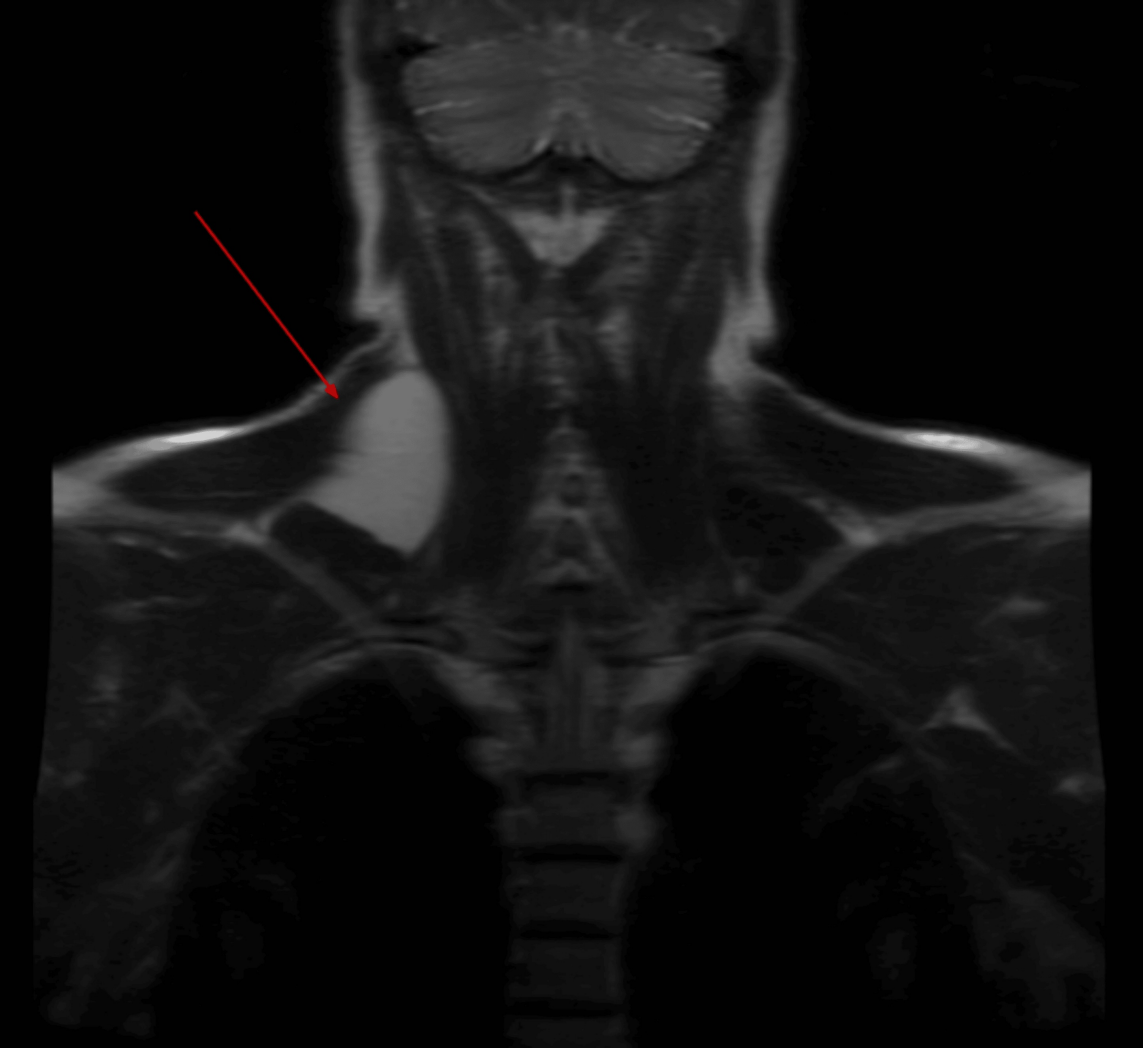
MRI cervical spine without contrast Deep right trapezius lipoma (red arrow) measuring 5.8 cm x 2.6 cm x 5.8 cm at the level of the cervicothoracic junction.

A 54-year-old right-hand dominant male presented in 2023 with a 12-year history of gradually worsening right anterolateral shoulder pain, rated 7/10 on the Visual Analog Scale. His discomfort was accompanied by periscapular pain, particularly aggravated by overhead activities. Electrodiagnostic studies (EDX) with electromyography (EMG) were conducted on January 22, 2024, which showed mild chronic C6-C7 radiculopathy. No signs of focal nerve entrapment, brachial plexopathy, peripheral neuropathy, or myopathy were identified (see Table 1). A magnetic resonance image (MRI) of the cervical spine without contrast on January 31, 2024, revealed a large, deep right trapezius lipoma measuring 5.8 cm x 2.6 cm x 5.8 cm at the cervicothoracic junction (see Figure 1).

**Table 1:** EMG results prior to trapezius lipoma resection EMG: Electromyography; Ins Act: Insertional activity; Fibs: Fibrillations; Psw: Positive sharp waves; Amp: Amplitude; Dur: Duration; Poly: Polyphasic; Recrt: Recruitment; Int Pat: Interference pattern

Side	Muscle	Nerve	Root	Ins Act	Fibs	Psw	Amp	Dur	Poly	Recrt	Int Patt	Comment
Right	Deltoid	Axillary	C5-6	Nml	Nml	Nml	Nml	Nml	0	Nml	Nml	Nml
Right	Biceps	Musculocutaneous	C5-6	Nml	Nml	Nml	Nml	>12ms	0	Nml	Nml	Nml
Right	Triceps	Radial	C6-7-8	Nml	Nml	Nml	Nml	>12ms	0	Nml	Nml	Nml
Right	Brachioradialis	Radial	C5-6	Nml	Nml	Nml	Nml	>12ms	0	Nml	Nml	Nml
Right	Extensor Indicis	Radial (Posterior Interosseous)	C7-8	Nml	Nml	Nml	Nml	Nml	0	Nml	Nml	Nml
Right	Flexor Carpi Radialis	Median	C6-7	Nml	Nml	Nml	Nml	>12ms	0	Nml	Nml	Nml
Right	Flexor Carpi Ulnaris	Ulnar	C8-T1	Nml	Nml	Nml	Nml	Nml	0	Nml	Nml	Nml
Right	Abductor Pollicus Brevis	Median	C8-T1	Nml	Nml	Nml	Nml	Nml	0	Nml	Nml	Nml
Right	1st Dorsal Interosseous	Ulnar	C8-T1	Nml	Nml	Nml	Nml	Nml	0	Nml	Nml	Nml
Right	Cervical Paraspinal Middle	Dorsal Rami	C4-6	Increased	Nml	Nml	Nml	Nml	0	Nml	Nml	Nml
Right	Trapezius	Spinal Accessory	CN XI, C3-4	Nml	Nml	Nml	Nml	Nml	0	Nml	Nml	Nml
Right	Supraspinatus	Suprascapular	C5-6	Nml	Nml	Nml	Nml	Nml	0	Nml	Nml	Nml
Right	Infraspinatus	Suprascapular	C5-6	Nml	Nml	Nml	Nml	Nml	0	Nml	Nml	Nml

In May 2024, the patient underwent elective surgical resection of the lipoma. Although the operative report was unavailable for review, the patient reported no known intraoperative complications. However, within a week after surgery, the patient began experiencing new symptoms, including paresthesias in the upper pectorals and trapezius regions, along with weakness during shoulder shrugging and abduction. These symptoms significantly restricted his ability to perform daily tasks and engage in recreational activities involving overhead movements.

Six months after lipoma resection, physical examination revealed mild atrophy of the right lower trapezius, with worsened myofascial trigger points in the scapulothoracic region. The surgical site showed a well-healing trapezius scar with hypertrophic changes, consistent with the location of the procedure. Active range of motion testing revealed new-onset scapular dyskinesis, with lateral scapular winging observed during active shoulder abduction at 100 degrees. Provocative testing revealed the following key findings: asymmetric weakness during resisted right shoulder shrug, lateral scapular winging, positive active external rotation test, and a positive wall push-up test.

Due to these new symptoms, repeat EDXs were performed on January 29, 2025. EMG of the right trapezius revealed increased insertional activity, prominent fibrillations (4+), widespread spontaneous activity, diminished recruitment, complex regional discharges (CRD), and a moderately decreased (75%) interference pattern (see Table 2). The right supraspinatus muscle demonstrated diminished recruitment and a reduced interference pattern, while all other muscles tested were unremarkable (see Table 2). In summary, the findings indicated severe focal neuropathy of the right SAN with ongoing denervation, evidenced by CRD in the trapezius and poor insertional patterns in the supraspinatus. The intact activation of the infraspinatus suggests that the issue was primarily muscular and related to the lipoma removal rather than an isolated nerve injury.

**Table 2:** EMG results after trapezius lipoma resection EMG: Electromyography; Ins Act: Insertional activity; Fibs: Fibrillations; Psw: Positive sharp waves; Amp: Amplitude; Dur: Duration; Poly: Polyphasic; Recrt: Recruitment; Int Pat: Interference pattern; CRD: Complex regional discharge

Side	Muscle	Nerve	Root	Insp Act	Fibs	Psw	Amp	Dur	Poly	Recrt	Int Patt	Comment
Right	Deltoid	Axillary	C5-6	Nml	Nml	Nml	Nml	Nml	0	Nml	Nml	Nml
Right	Biceps	Musculocutaneous	C5-6	Nml	Nml	Nml	Nml	Nml	0	Nml	Nml	Nml
Right	Triceps	Radial	C6-7-8	Nml	Nml	Nml	Nml	Nml	0	Nml	Nml	Nml
Right	Brachioradialis	Radial	C5-6	Nml	Nml	Nml	Nml	Nml	0	Nml	Nml	Nml
Right	Flexor Carpi Radialis	Median	C6-7	Nml	Nml	Nml	Nml	Nml	0	Nml	Nml	Nml
Right	Flexor Carpi Ulnaris	Ulnar	C8-T1	Nml	Nml	Nml	Nml	Nml	0	Nml	Nml	Nml
Right	1st Dorsal Interosseous	Ulnar	C8-T1	Nml	Nml	Nml	Nml	Nml	0	Nml	Nml	Nml
Right	Abductor Pollicus Brevis	Median	C8-T1	Nml	Nml	Nml	Nml	Nml	0	Nml	Nml	Nml
Right	Trapezius	Spinal Accessory	CN XI, C3-4	Increased	4+	Nml	Nml	Nml	0	Reduced	75%	CRD
Right	Rhomboid Major	Dorsal Scapular	C5	Nml	Nml	Nml	Nml	Nml	0	Nml	Nml	Nml
Right	Rhomboid Minor	Dorsal Scapular	C5	Nml	Nml	Nml	Nml	Nml	0	Nml	Nml	Nml
Right	Supraspinatus	Suprascapular	C5-6	Nml	Nml	Nml	Nml	Nml	0	Reduced	25%	Nml
Right	Infraspinatus	Suprascapular	C5-6	Nml	Nml	Nml	Nml	Nml	0	Nml	Nml	Nml
Right	Latissimus Dorsi	Thoracodorsal	C6-8	Nml	Nml	Nml	Nml	Nml	0	Nml	Nml	Nml
Right	Serratus Anterior	Long Thoracic	C5-7	Nml	Nml	Nml	Nml	Nml	0	Nml	Nml	Nml

This patient was subsequently referred to a peripheral nerve surgeon, with clinical recommendations for conservative versus procedural considerations pending.

## Discussion

SAN mononeuropathy is an uncommon but significant complication following surgical procedures in the posterior cervical and shoulder region. While it has been well-documented in the context of lymph node biopsies, radical neck dissections, and penetrating trauma, its occurrence after lipoma resection is yet to be published. To the best of our knowledge, this case represents the first reported instance of isolated SAN injury following trapezius lipoma resection, highlighting not only the importance of this potential complication, but also the importance of early recognition and intervention.

Mechanism of injury

The SAN has a particularly vulnerable anatomic course, traversing the posterior triangle of the neck before penetrating the deep surface of the trapezius muscle [1,2]. On average, the SAN runs 2.67 cm beneath the skin, 3.80 cm from the vertebral spinous process, and only 0.70 cm from the medial border of the scapula, making it particularly susceptible during posterior cervical surgeries [8]. In this case, the resection of a deeply situated trapezius lipoma likely led to indirect SAN injury through excessive traction, compression, or thermal damage rather than direct transection, as no intraoperative complications were noted [9].

Clinically, SAN injury manifests with characteristic signs, including lateral scapular winging, vague shoulder pain, muscle atrophy, and functional limitations in shoulder elevation and abduction [6]. These findings were evident in the patient in this report within a week postoperatively, emphasizing the importance of early recognition. EDX confirmed severe focal SAN mononeuropathy with ongoing denervation, reinforcing the vulnerability of this nerve in the posterior cervical triangle.

Clinical and functional implications

SAN injury often results in scapular dyskinesis, leading to abnormal scapulohumeral rhythm, reduced shoulder stability, and compensatory overuse of surrounding musculature. This can lead to chronic pain and functional impairment, particularly in overhead activities [6,8]. The persistence of denervation findings on follow-up EMG six months postoperatively suggests a more severe nerve injury that may not fully recover without intervention.

The literature suggests that functional recovery following SAN injury is time-sensitive. Outcomes vary widely, with better prognosis if surgical repair is performed within seven months of injury, whereas delayed interventions beyond 20 months have progressively worse results [6,7].

Management considerations

The management of iatrogenic SAN injury varies depending on the severity and chronicity of the condition. Early identification is crucial, as timely intervention can improve functional outcomes. Treatment for SAN mononeuropathy ranges from conservative rehabilitation to surgical intervention. Physical therapy focusing on scapular stabilization, neuromuscular re-education, and myofascial release can be effective in mild to moderate cases, especially if nerve regeneration occurs spontaneously [7,10]. However, in this case, the patient’s persistent weakness and progressive symptoms beyond the expected recovery period suggest a more severe injury - potentially warranting surgical exploration of nerve reconstruction [7,11,12].

Given the severity of this case, surgical interventions such as nerve grafting, neurolysis, or dynamic tendon transfers may be considered. Tendon transfers, particularly Eden-Lange procedures or levator scapulae-rhomboid transfers, have been described for cases of persistent trapezius paralysis with significant scapular winging [13,14].

The only limitation of this report is the lack of intraoperative details, making it difficult to determine the precise mechanism of nerve injury. Further studies are needed to establish the best practices for preventing and managing SAN injuries in similar cases.

## Conclusions

This case highlights the significant risk of SAN injury following trapezius lipoma resection. The patient’s postoperative symptoms, including scapular dyskinesis and persistent weakness, emphasize the importance of early recognition and diagnostic confirmation through EDX. Given the evidence of ongoing denervation, conservative management alone is insufficient, warranting consideration of surgical interventions such as nerve grafting or tendon transfers. This report underscores the necessity of meticulous surgical technique to minimize iatrogenic SAN injury and the critical role of timely intervention in optimizing patient outcomes. This case was selected due to its unique presentation of isolated SAN injury following trapezius lipoma resection. This is a complication not previously documented in the literature. Given the SAN's anatomical vulnerability in the posterior cervical triangle, this case provides valuable insight into an under-recognized surgical risk. The findings contribute to the growing awareness of nerve preservation strategies and highlight the importance of early diagnosis and intervention to optimize patient outcomes. By sharing this case, we aim to inform physicians about the potential for SAN injury in similar procedures.
